# Complex Inheritance of Melanoma and Pigmentation of Coat and Skin in Grey Horses

**DOI:** 10.1371/journal.pgen.1003248

**Published:** 2013-02-07

**Authors:** Ino Curik, Thomas Druml, Monika Seltenhammer, Elisabeth Sundström, Gerli Rosengren Pielberg, Leif Andersson, Johann Sölkner

**Affiliations:** 1 Department of Animal Science, Faculty of Agriculture, University of Zagreb, Zagreb, Croatia; 2 Department of Sustainable Agricultural Systems, University of Natural Resources and Applied Life Sciences, Vienna, Austria; 3 Department of Vascular Biology and Thrombosis Research, Medical University of Vienna, Vienna, Austria; 4 Science for Life Laboratory, Department of Medical Biochemistry and Microbiology, Uppsala University, Uppsala, Sweden; 5 Department of Animal Breeding and Genetics, Swedish University of Agricultural Sciences, Uppsala, Sweden; University of Melbourne, Australia

## Abstract

The dominant phenotype of greying with age in horses, caused by a 4.6-kb duplication in intron 6 of *STX17*, is associated with a high incidence of melanoma and vitiligo-like skin depigmentation. However, the progressive greying and the incidence of melanoma, vitiligo-like depigmentation, and amount of speckling in these horses do not follow a simple inheritance pattern. To understand their inheritance, we analysed the melanoma grade, grey level, vitiligo grade, and speckling grade of 1,119 Grey horses (7,146 measurements) measured in six countries over a 9-year period. We estimated narrow sense heritability (h^2^), and we decomposed this parameter into polygenic heritability (h^2^_POLY_), heritability due to the Grey (*STX17*) mutation (h^2^_STX17_), and heritability due to agouti (*ASIP*) locus (h^2^_ASIP_). A high heritability was found for greying (h^2^ = 0.79), vitiligo (h^2^ = 0.63), and speckling (h^2^ = 0.66), while a moderate heritability was estimated for melanoma (h^2^ = 0.37). The additive component of *ASIP* was significantly different from zero only for melanoma (h^2^_ASIP_ = 0.02). *STX17* controlled large proportions of phenotypic variance (h^2^_STX17_ = 0.18–0.55) and overall heritability (h^2^_STX17_/h^2^ = 0.28–0.83) for all traits. Genetic correlations among traits were estimated as moderate to high, primarily due to the effects of the *STX17* locus. Nevertheless, the correlation between progressive greying and vitiligo-like depigmentation remained large even after taking into account the effects of *STX17*. We presented a model where four traits with complex inheritance patterns are strongly influenced by a single mutation. This is in line with evidence of recent studies in domestic animals indicating that some complex traits are, in addition to the large number of genes with small additive effects, influenced by genes of moderate-to-large effect. Furthermore, we demonstrated that the *STX17* mutation explains to a large extent the moderate to high genetic correlations among traits, providing an example of strong pleiotropic effects caused by a single gene.

## Author Summary

Clarifying the genetic architecture of complex traits is a problem with profound implications for agriculture, biology, and medicine. Using data from Lipizzan horses with the grey coat phenotype, we present an example of a single mutation (intronic duplication in *STX17*) that explains 18%–55% of phenotypic variation in four complex traits, while polygenic background additive effects also explain 11%–57% of phenotypic variation. This study provides a prime example of complex traits being influenced by genes of moderate-to-large effect and supports further the evidence of recent studies in domestic animals that some complex traits are, in addition to the large number of genes with small additive effects, influenced by genes of moderate-to-large effect. We further show that the *STX17* mutation accounts for a large proportion of the estimated genetic correlations between the traits. This case of strong pleiotropic effects of a single mutation on complex traits makes this work of significant general interest for biology and medicine.

## Introduction

Recent developments in molecular genetics have enabled molecular dissection of quantitative traits in humans [1], model organisms [2] and domestic animals [3]. Genome-wide association (GWA) studies suggest that variability of complex traits is caused by many loci, most exerting tiny effects, whereas loci exerting moderate-to-large effects or loci that explain more than 5–10% of phenotypic variation are rare [3],[4],[5]. In human populations, those genes with moderate-to-large effects do appear in low frequency as rare or “private” mutations [6]. In contrast, appearance of moderate-to-large effect mutations at intermediate or high frequencies is documented in domestic animal populations, perhaps, as consequence to change in selection pressure caused by domestication [7].

The genetic variation of several complex traits in Grey horses is considerably affected by at least one gene of moderate-to-large effect. Grey horses are born with their base colour (e.g. black, bay, chestnut), which then greys early in life due to loss of melanocytes, a process similar to the greying of hair in humans, which typically occurs much later in life. The mode of inheritance of the greying phenotype is autosomal dominant. A grey horse will be either *GG* or *Gg*, non-grey horses carry the *gg* genotype. Previously we reported that greying with age is caused by a 4.6-kb duplication in intron 6 of *STX17*, which encodes syntaxin 17 [8]. In addition to grey level, the Grey mutation was found to strongly influence melanoma, vitiligo and speckling grade as well as to capacitate the effects of the loss-of-function mutation at the agouti (*ASIP*) locus on melanoma grade. Recently, we have shown that the duplicated region contains a melanocyte-specific enhancer that becomes much stronger when duplicated [9]. Examination of melanoma tissue revealed copy number variation of the 4.6 kb intron in STX17, with a difference between blood and tumor DNA. The highest copy numbers occurred in tumors classified as aggressive [10]. Grey horses are the only animals to present progressive coat greying, melanoma, vitiligo-like skin depigmentation and coat speckling, making them an excellent model for studying the inheritance of traits with complex genetic background.

Studies of coat colour in horses led to the definition of discrete phenotypes (e.g. bay, black, brown, grey) controlled by a few genes showing epistatic interaction [11]. This typology, although practical for breeders to some extent, could not account for variation in greying level. Curik et al. [12] provided a quantitative description of progressive greying, while Toth et al. [13] found relationship between that quantitative greying measure and total melanin content of horse hair.

Melanoma occurs frequently in grey horses, in three histopathologically defined clinical patterns. In the first pattern, which describes the majority of cases, the melanoma grows slowly over many years without evidence of regional or distant metastases. In the second pattern, the melanoma results from malignant transformation of a benign melanoma (melanocytoma; [14]). In the third pattern, which is rare, the melanoma is malignant from onset [15]. Although most melanomas in Grey horses present benign features at diagnosis, nearly 66% become malignant later [16]. Melanoma in Grey horses occurs most frequently underneath the tail, in the peri-anal region, and around the lips and eyelids [17],[18]. The frequency of melanoma occurrence is around 80% in horses older than 15 years [19].

The hereditary component of melanoma in Grey horses was first studied by Rieder et al. [20]. Using segregation analysis, they were unable to establish whether the mode of inheritance was monogenic, polygenic or mixed, because of the relatively low number of horses examined (n = 71). Nevertheless, models including a polygenic component fitted the data significantly better than did a non-genetic model. In a more recent study involving 296 grey Lipizzan horses, Seltenhammer et al. [21] estimated a heritability of 0.36 for melanoma grade.

Grey horses also show vitiligo-like depigmentation. In fact, Seltenhammer et al. [21] found equine vitiligo in 50% of older grey Lipizzaner horses. In humans, vitiligo is a chronic disorder characterized by sharply delimited, progressive, patchy loss of pigmentation resulting from death or dysfunction of the cells responsible for skin and hair pigmentation [22]. While the pathogenesis of human vitiligo is unknown, it is considered a complex hereditary disease, and several theories have been proposed to explain it [23],[24]. Similarly, although linked to progressive greying, the pathogenesis of equine vitiligo remains largely unknown. Like melanoma, vitiligo occurs far more frequently in Grey horses than for those with solid colours, indicating that it has a hereditary component that may be associated with progressive greying. Although rare, studies in humans seem to indicate that melanoma and vitiligo might be genetically linked [25],[26].

When this study was conceived, we hypothesized that while the effects of *STX17* are large for all four traits and ASIP has a considerable effect of melanoma, a large part of the genetic variation remains unexplained. We further hypothesized that the genetic correlations among those traits are strongly influenced by *STX17*. Here we have extended our previous studies by increasing the number of genotyped horses and parameters examined, including genotype at the *STX17* and *ASIP* loci. Firstly, for all four traits (melanoma, grey level, vitiligo, speckling), we decomposed estimated repeatability into a component due to permanent environmental effects and a component describing narrow sense heritability. We further decomposed estimated narrow sense heritability into a component describing background polygenic heritability and a components describing heritability caused by *STX17* and *ASIP*. We also estimated genetic correlations among all four traits using various models designed to evaluate the contribution from *STX17* and *ASIP* genes and the contribution from pleiotropy due to additional polygenic background versus that of *STX17* and *ASIP* genes. Finally, we graphically illustrated the genetic relationship among melanoma grade, grey level, vitiligo grade and speckling grade with respect to *STX17* genotype.

## Results

Estimated fixed effects and variance components for melanoma grade, grey level, vitiligo grade and speckling grade are shown in Table 1, together with means and standard errors. Linear effects of age were significant (P<0.001) for all four traits. Thus, for example, a 6-year-old grey horse will present a grey colour that is 5.88 L* units darker than that of a 7-year-old. Linear regression coefficients relating each trait to *STX17* were highly significant (P<0.001) for all traits studied, while the corresponding coefficients involving *ASIP* were significant only for melanoma grade (P<0.001). In quantitative genetics, estimated linear regression coefficients can be interpreted as gene substitution effects of *STX17* (α_STX17_) and *ASIP* (α_ASIP_). Thus, the regression coefficient of −13.78 for the Grey mutation in relation to the trait Greying indicates that heterozygotes *Gg* on average will be 13.78 L* units darker than homozygous *GG* horses. *ASIP* has three genotypes, so the melanoma grade is expected to change by 0.38 units (2α_ASIP_) when moving from an *AA* to an *aa* genotype.

**Table 1:** Estimated fixed effects and variance components* for melanoma grade, grey level, vitiligo grade, and speckling grade.

Parameter	Melanoma	Greying	Vitiligo	Speckling
Mean for 7-year-old horses	0.56±0.81	70.15±8.89	0.85±0.85	0.39±0.72
Horses/Observations	516/1310	541/831	447/789	441/724
Fixed effects				
Age at measurement (months by 12)	0.11±0.01	5.88±0.24	0.06±0.02	0.02±0.01
Grey mutation (α_STX17_; *GG* = 0, *Gg* = 1)	−0.85±0.08	−13.78±1.16	−0.82±0.08	1.36±0.06
Agouti gene (α_ASIP_; *AA* = 0, *Aa* = 1, *aa* = 2)	0.19±0.06	n.s.**	n.s.	n.s.
Random effects				
Additive polygenic variance (V_POLY_)	0.19±0.07	99.19±19.26	0.29±0.07	0.09±0.04
Permanent environment variance (V_pe_)	0.27±0.06	5.06±11.50	0.00±0.05	0.10±0.03
Residual error variance (V_e_)	0.39±0.02	32.66±2.77	0.26±0.02	0.19±0.02

* Results were obtained from the univariate models (see Materials and Methods). Estimates related to the stud by sex by year of measurement were significant but are not presented here as there were a large number of class effects.

** n.s. = Non-significant.

The frequency of the Grey allele was between 0.85 and 0.89 for the various data sets. Univariate estimates were obtained for heritability (h^2^) and for repeatability (R), which here corresponds to the upper bound of broad sense heritability, together with estimates of phenotypic variance (V_P_) and variance due to permanent environmental effects (c^2^), here variance introduced by repeated measurement (Table 2). Proportions of variance in grey level and vitiligo grade due to permanent environmental effects were 0.03 and 0.00, respectively, whereas these proportions were much larger for melanoma grade (0.26) and speckling grade (0.12). Very high h^2^ values, that explained 63–79% of the phenotypic variance, were estimated for grey level, vitiligo grade and speckling grade. In contrast, moderate h^2^ was estimated for melanoma grade. Across all models standard errors of hertiability estimates ranged from 0.08 to 0.12. For each trait we decomposed h^2^ into h^2^_POLY_, h^2^_STX17_ and h^2^_ASIP_ (melanoma grade) (Table 1). The polygenic component was highest for grey level (h^2^_POLY_ = 0.57), followed by the estimated components for vitiligo grade (h^2^_POLY_ = 0.41), melanoma grade (h^2^_POLY_ = 0.18) and speckling grade (h^2^_POLY_ = 0.11). The heritability caused by the *STX17* mutation was highest for speckling grade (h^2^_STX17_ = 0.55) and moderate for vitiligo grade (h^2^_STX17_ = 0.23), grey level (h^2^_STX17_ = 0.22) and melanoma grade (h^2^_STX17_ = 0.18). The additive component of *ASIP* was significantly different from zero only for melanoma grade (h^2^_ASIP_ = 0.02). When polygenic heritability was assessed only for homozygous *GG* horses, obtained values were still high for all four traits: grey level, 0.76; melanoma, 0.30; vitiligo, 0.57; and speckling, 0.36. An analysis of this data set for melanoma including ASIP revealed no substantial increase of heritability (h^2^_ASIP_ = 0.03) while the effect of *ASIP* on other traits remained non-significant.

**Table 2:** Phenotypic variance, repeatability, and heritabilities for melanoma grade, grey level, vitiligo grade, and speckling grade.*

Genetic parameter**	Melanoma	Greying	Vitiligo	Speckling
Phenotypic variance (V_P_)	1.05	175.45	0.72	0.83
Repeatability (R)	**0.63**	**0.81**	**0.64**	**0.77**
Explained permanent environment (c^2^)	0.26	0.03	0.00	0.12
Narrow sense total heritability (h^2^)	**0.37**	**0.79**	**0.64**	**0.66**
Pure polygenic heritability (h^2^_POLY_)	0.18	0.57	0.41	0.11
Grey mutation heritability (h^2^_STX17_)	0.18	0.22	0.23	0.55
Agouti gene heritability (h^2^_ASIP_)	0.02	0.00	0.00	0.00

* Results were obtained from the univariate models (see Materials and Methods).

** Genetic parameters were calculated as follows: V_P_ = V_POLY_+V_STX17_+V_ASIP_+V_pe_+V_e_; R = (V_POLY_+V_STX17_+V_ASIP_+V_pe_,)/V_P_; c^2^ = V_pe_/V_P_; h^2^ = (V_POLY_+V_STX17_+V_ASIP_)/V_P_; h^2^_POLY_ = V_POLY_/V_P_; h^2^_STX17_ = V_STX17_/V_P_; h^2^_ASIP_ = V_ASIP_/V_P_. For all traits except melanoma, we assumed V_ASIP_ = 0. The frequencies ranged from 0.85 to 0.89 for the *STX17* G allele and from 0.51 to 0.53 for the *ASIP* A allele in the various data sets.

**Figure 1:**
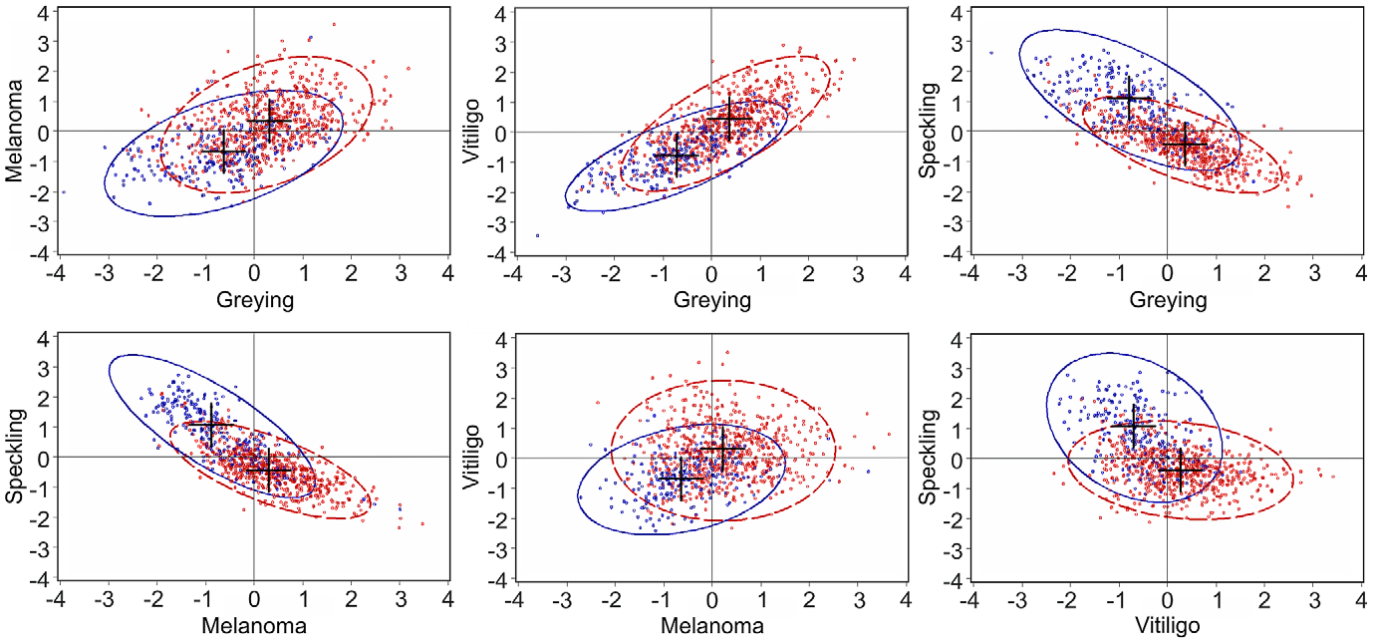
Scatter plots of standardized estimated breeding values (EBVs) from bivariate analysis (first scenario). EBVs were corrected for age and sex by stud by year effect. Homozygous (*GG*) animals are shown in red; heterozygous (*Gg*) animals, in blue. Contour plot ellipses contain 95% of the data.

Multivariate models were used to estimate phenotypic (r_P_) and genetic correlations (r_POLY_) according to three scenarios (Table 3). In the first scenario, we estimated genetic correlations with a standard polygenic model, ignoring the effects of *STX17* and *ASIP*. In the second scenario, we used a model that includes the fixed effects of these two mutations. For the third scenario, we applied a polygenic model only to the data for *GG* horses. In the first scenario, the genetic correlations among the traits followed the same pattern as the phenotypic ones, with the genetic values occasionally somewhat higher (e.g. 0.67 vs. 0.52 in the case of grey level and vitiligo grade). When the genetic effects were included (second scenario) or when only homozygous grey horses were considered (third scenario), correlations dropped substantially. This is a strong indication of the pleiotropic effects of the *Grey* mutation. Only the genetic relationship between grey level and vitiligo grade remained significant and considerable across all three scenarios (0.67±0.06 in the first, 0.48±0.09 in the second, and 0.50±0.10 in the third). Visualization of the genetic correlations due to polygenic effects is presented by scatter plots of estimated breeding values (Figure 1). Distances from a homozygous (*GG*) genotype of *STX17* to a heterozygous one (*Gg*) have a diagonal shift in the contour plot centres. In addition to these pleiotropic effects of the *STX17* mutation, the fact that the contour plots for breeding values were elliptical specifically for the *GG* and *Gg* genotypes indicates that genetic correlations due to polygenic background additive effects remained even after accounting for the *STX17* mutation. This was particularly obvious for the genetic correlation between grey level and vitiligo grade, for which the estimated genetic correlation after accounting for major genes remained significant (r_POLY_ = 0.48±0.09).

**Table 3:** Phenotypic (r_P_) and genetic correlations from polygenic components (r_POLY_) for melanoma grade, grey level, vitiligo grade, and speckling grade estimated using different scenarios.*

Trait pair	First scenario*		Second scenario	Third scenario
	r_P_	r_POLY_	r_POLY_	r_POLY_
Melanoma, Greying	0.33±0.12	0.39±0.12	0.05±0.16	0.02±0.16
Melanoma, Vitiligo	0.22±0.04	0.28±0.17	−0.19±0.23	−0.09±0.14
Melanoma, Speckling	−0.43±0.04	−0.70±0.11	−0.28±0.26	−0.15±0.26
Greying, Vitiligo	0.52±0.05	0.67±0.06	0.48±0.09	0.50±0.10
Greying, Speckling	−0.34±0.06	−0.41±0.16	0.00±0.16	−0.18±0.16
Vitiligo, Speckling	−0.43±0.04	−0.47±0.14	0.00±0.23	0.01±0.24

* The first scenario is a polygenic model applied to data from all genotyped horses. The second scenario is a polygenic model that also includes *STX17* (for all traits) and *ASIP* (only for melanoma grade), applied to data from all genotyped horses. The third scenario is the polygenic model applied to data only from homozygous (*GG*) horses, corresponding to 70–73% of the complete data set, depending on the trait.

## Discussion

We present a case in which the genetic components of four complex traits could be decomposed into the effects of polygenic additive effects and the monogenic effects of *STX17* and *ASIP* mutations. The data available was still insufficient to allow accurate estimation of polygenic dominance variance or of higher-order, non-additive genetic variances. Although melanoma and vitiligo have been intensively studied in humans, few estimates of their quantitative inheritance are available. The present study provides some of the first quantitative insights into their inheritance in grey horses. While *STX17* explains large proportions of the phenotypic variance, we were surprised to see that, when only the data from homozygous *GG* horses were analysed, the residual polygene component still explained a large part of the variation in melanoma and vitiligo grade (Table 3). These results argue for the need to search for more genes involved in the expression of these traits.

The *STX17* mutation accounts for considerable amount of the estimated genetic correlations among all traits analysed; in other words, it exhibits strong pleiotropic effects. While precise estimates of genetic correlation require extremely large data sets, our findings are supported by scatter plots of estimated breeding values (Figure 1). Graphical illustrations also indicated that polygenic additive effects were causing negative genetic correlations between melanoma grade and speckling grade as well as between grey level and speckling grade. However, we were not able to confirm those evidences numerically since related estimates of genetic correlations had very wide confidence intervals (Table 3). On the other side, we were able to show that estimated genetic correlation between melanoma and vitiligo was negligible after accounting for the pleiotropic effect of *STX17* mutation (Table 3 and Figure 1). However, it is highly speculative to conclude the same pattern, just one gene linking melanoma and vitiligo, is applying to humans.

The results obtained here, further, support the evidence of recent studies in domestic animals that some complex traits are, in addition to the large number of genes with small additive effects, influenced by genes of moderate-to-large effect at intermediate to high frequencies. For example, a single nucleotide substitution in intron 3 of *IGF2* in pigs explain about 30% of the residual phenotypic variance for lean meat in ham in a wild boar/Large White intercross [27],[28]. Grisart et al. [29] have also shown that the mutation in *DGAT1*, appearing in different breeds at varying gene frequencies, considerably decreases milk fat percentage (h^2^_DGAT1_ = 0.51; h^2^_POLY_ = 0.29) and yield (h^2^_DGAT1_ = 0.15; h^2^_POLY_ = 0.55) while increases milk protein yield (h^2^_DGAT1_ = 0.08; h^2^_POLY_ = 0.65) and milk volume h^2^_DGAT1_ = 0.18; h^2^_POLY_ = 0.49). A strong effect of *PLAG1* mutation (h^2^_PLAG1_ = 0.07) on the postpartum calf weight was recently estimated in the outbreed dairy cattle population [30]. Moderate-to large effects have been also demonstrated for the myostatin mutation on the meat and carcass quality in beef cattle [31]. The genetic architecture of complex traits presented here, in which quantitative variation is explained by a strong contribution from a single gene and/or mutation together with a substantial polygenic additive component, may be frequent in traits related to colouration and pigmentation of coat and skin as successful adaptation requires fast and slow responses. A similar genetic architecture was presented by Hayes et al. [32] in a study where *KIT*, *MITF*, and a locus on chromosome 8 together explained 24% of the variation in the proportion of black coat in cattle. Recently, Liu et al. [33] also indicated that a few genes played a major role in human eye colour. Coat and skin colouration, the consequences of melanin-dependent pigmentation, are evolutionarily important traits because they are involved in several aspects of survival, such as hiding from predators and thermoregulation [34]. Fang et al. [35] concluded that coat colour variation in domestic animals has been shaped by artificial selection. The increase in the frequency of mutated allele G, here up to 89%, is strongly associated with human appreciation of grey colour in horses. The high incidence of melanoma and vitiligo in Grey horses is most likely the result of negative pleiotropic effects of the mutations contributing to the grey phenotype, in particular the Grey mutation itself. The presence of this prime example provides, thus, an additional insight into genetic architecture of complex traits and their biology.

## Materials and Methods

### Animals and data collection

Data were recorded from grey Lipizzan horses situated at six state-owned studs from Austria (Piber), Bosnia and Herzegovina (Vucjak), Croatia (Djakovo), Hungary (Szilvasvarad), Slovakia (Topol'cianky) and Slovenia (Lipica). Studs were visited repeatedly over nine years (1999–2007) and a total of 7146 measurements were collected from 1119 horses. All horses analysed were connected by a complete pedigree that includes 4961 members and extends back to the 18th century. Most data came from the offspring of 189 sires (mean, 5.97; maximum, 33) and 496 dams (mean, 2.26; maximum, 11).

### Quantitative description of the melanoma phenotype

**Figure 2:**
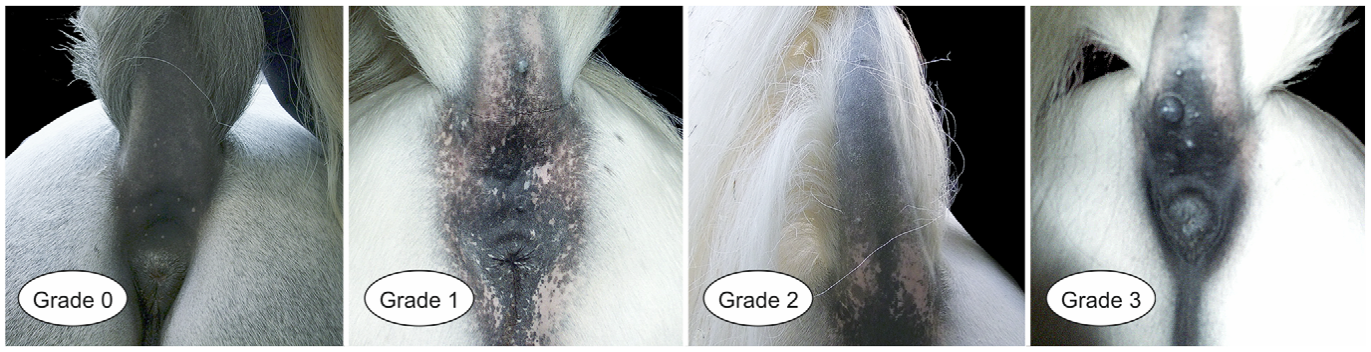
Variation of melanoma grade in four grey Lipizzan horses.

Melanoma was detected by adspection and palpation. Melanoma grade was defined according to a modified classification system on a scale from 0.0 to 5.0 that allows for intermediate grades (e.g. 0.0, 0.5, 4.5), described in [17] (Table 4). Adspection was conducted at sites melanomas typically occur, such as the peri-anal and anal region, the perineal region, udder, and praeputium. The tail was bent upwards in order to detect even the smallest, plaque-like lesions. Lips and eyelids, the parotis, the peri-ocular region and ears were also examined. Finally, the whole integument was checked for potential tumours. Variation of melanoma grade in four grey Lipizzan horses is illustrated in Figure 2.

**Table 4:** Clinical classification of melanoma grade in grey Lipizzan horses.

Grade	Description
0	Free of melanoma
1	Early stages of plaque-type or one solitary nodus with a diameter of 0.5 cm at typical locations
2	Several nodules with a diameter of 0.5 cm, or one solitary nodus with a diameter of 2 cm, at typical locations
3	One or several nodular melanomas with a diameter of 5 cm intra- and/or subcutaneous at typical locations (or lips)
4	Extensive confluent melanoma covered with skin; signs of destruction (necrosis, ulceration); metastasis
5	Exophytic growth of tumours, which show wet surfaces and ulceration; metastasis into different organs accompanied by paraneoplastic syndrome (cachexia, fever, metabolic disorders)

### Quantitative description of the progressive greying phenotype

**Figure 3:**
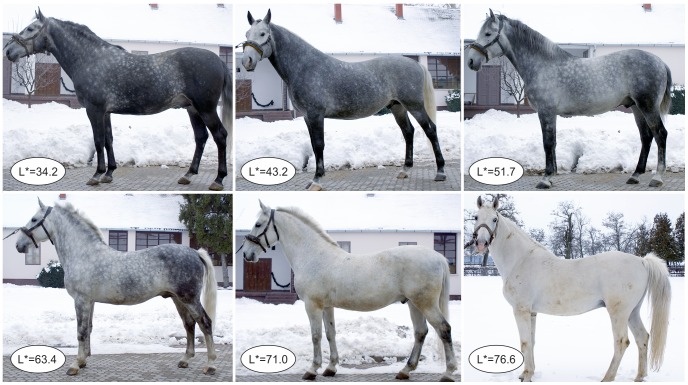
Variation of grey levels of the coat, represented by L* values, in six grey Lipizzan horses.

Progressive greying was quantified with a Minolta Chromameter CR210 using the CIE L*a*b* colour space. In this system, colour is quantified by its reflection along three axes: white-black (L*), red-green (a*) and yellow-blue (b*). We referred only to the L* parameter defined on a scale from 0 (black - total absorption) to 100 (white - total reflection). Various grey levels in seven Lipizzan horses are illustrated in Figure 3. Each horse was measured at four places (neck, shoulder, belly and croup), and the average of the four measurements was used to represent the grey level.

### Quantitative description of the vitiligo phenotype

**Figure 4:**
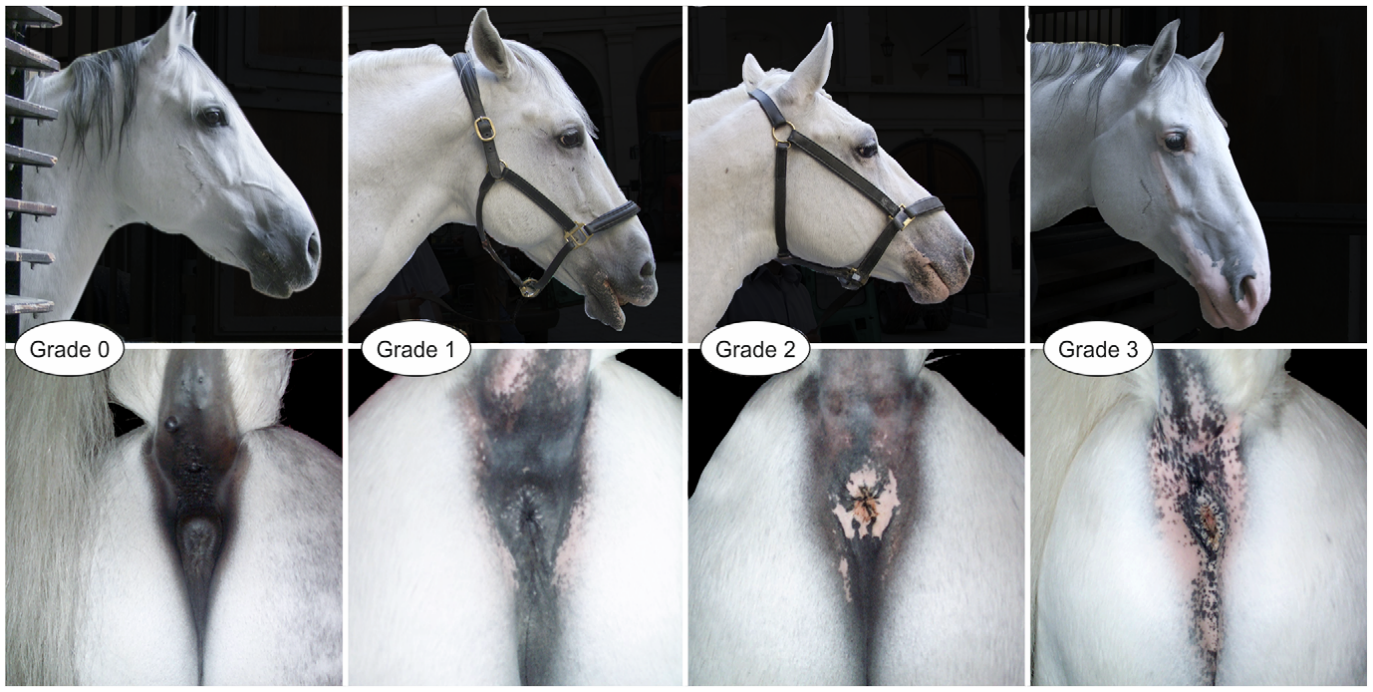
Variation of vitiligo grade in four grey Lipizzan horses.

Vitiligo was graded on a scale from 0 (no vitiligo) to 3 (severe vitiligo) based on adspection of typical sites, such as the peri-anal and anal region, the perineal region, udder, praeputium and the face, especially around the nostrils and eyes. Vitiligo grade was evaluated simultaneously with melanoma. The overall vitiligo grade was the average of included vitiligo grades from patches in the perianal and the facial regions. Variation of vitiligo grade across four grey Lipizzan horses is illustrated in Figure 4.

### Quantitative description of the speckling phenotype

**Figure 5:**
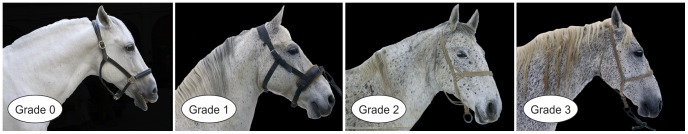
Variation of speckling grade in four grey Lipizzan horses.

Adult grey horses may present coloured specks or spots on a grey background. The amount of speckling was graded on a scale from 0 (not speckled) to 3 (heavily speckled). Variation in speckling grade across four grey Lipizzan horses is illustrated in Figure 5.

### Genotyping

Genotyping for the *STX17* mutation was carried out on 760 horses and for the *ASIP* gene on 667 horses; the *agouti* locus controls the inheritance of black and bay base colour [36]. Genotyping procedures were performed as described in Pielberg et al. [8]. The genotypes of additional horses were deduced by combining genotyping and pedigree information. For instance, a single coloured offspring qualifies a grey parent as heterozygous, grey offspring with one coloured parent must be heterozygous. In this way we extended the number of genotyped horses to 966 for the *STX17* mutation and 873 for the *ASIP*. However, deducing genotypes may introduce bias in the estimation of major gene effects. To avoid potential bias, we performed analyses on data sets with and without deduced genotypes. The results were very similar, those presented here are therefore based on data sets containing deduced genotypes.

### Estimating (co)variance components and gene effects

All traits analysed (grey level and grades of melanoma, vitiligo and speckling) strongly depend on the age of the horse. Grey level can vary widely in animals during the first 6–10 years; from 10 years onwards, this variation drops considerably and all horses reach their final coat colour. Both melanoma and vitiligo show an age of onset of 6–8 years, though they do appear earlier in rare cases. Speckling has been defined only for adult horses, because the density of spots or speckles is difficult to assess while horses are still dark. Thus, we analysed grey level data only for horses seven years old and younger. Conversely, data on melanoma, vitiligo and speckling grade were used only for horses seven years old and older. The dynamics of melanoma, grey level, vitiligo and speckling with respect to age has been shown in Pielberg et al. [8].

Statistical analyses were performed with univariate and multivariate general mixed linear models using the ASReml package (version 3; [37]). These models are known as (Individual) Animal models; see [38] for more detailed explanation. For all of the variables analysed, the statistical models showed no serious statistical violations; residuals from the models followed a normal distribution and variances were roughly homogeneous. The following modeling strategy was applied. First, we fitted a model that included the effects of the stud by sex by year of measurement (fixed), age at measurement in months (covariate), *STX17* mutation effects (indicator variables, with *GG* = 0 and *Gg* = 1 treated as covariates), *ASIP* additive effects (indicator variables, with *AA* = 0, *Aa* = 1 and *aa* = 2 treated as covariates) and *ASIP* dominance effects (indicator variables, with *AA* = 0, *Aa* = 1 and *aa* = 0 treated as covariates) and all two-factor interactions. Two factor interactions and the ASIP dominance effects were non-significant for any trait and were dropped from further analyses. Initial and all subsequent models also included additive genetic animal effects (random) and permanent environmental effects (random), since measurements were taken repeatedly.

The following final model was used to analyse the data:
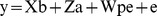
where the vector y represents the phenotypic values; b is the vector of fixed effects; a, pe and e are vectors of, respectively, polygenic additive effects (breeding values), permanent environmental (repeated measurement) effects and residual error values; X is the design matrix for fixed effects; and Z and W are design matrices for random effects. For all four traits analysed, the final models accounted for the stud by sex by year of measurement effect, for the effect of age at measurement and for the effect of *STX17*, while the effect of *ASIP* was also accounted for only in the case of melanoma grade. In addition, additive polygenic (V_POLY_), permanent environmental (V_pe_) and residual variances (V_e_), respectively, were defined as
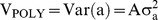


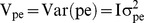


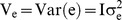
where A is an additive genetic relationship matrix and I is an identity matrix. In multivariate models we respected the modeling results obtained in univariate models.

Based on variance components and effects estimated from statistical analyses, together with allele frequencies obtained by counting, we derived quantitative genetic parameters to explain the complex inheritance of the traits analysed. Additive genetic variances of *STX17* mutation (V_STX17_) and *ASIP* (V_ASIP_) were calculated as V_STX17_ or V_ASIP_ = 2pqα^2^, where estimated linear regression coefficients are equal to gene substitution effects α (α = a for *STX17* and α = a+(p−q)d for *ASIP*), p and q are allele frequencies of *STX17* or/and *ASIP*, a is an additive value and d is a dominance value. Calculation of single gene (mutation) variances enabled us to calculate additive genetic variance as a sum of V_POLY_, V_STX17_ and V_ASIP_, and phenotypic variance as a sum of V_POLY_, V_STX17_, V_ASIP_, V_pe_, and V_e_. In further analysis, we calculated proportions of genetically explained variations from the phenotypic variance as follows: repeatability, defined as correlation among observations within individuals that provides an upper limit to broad sense heritability, R = (V_POLY_+V_STX17_+V_ASIP_+V_pe_,)/V_P_; permanent environmental effects, c^2^ = V_pe_/V_P_; narrow sense heritability, h^2^ = (V_POLY_+V_A-STX17_+V_A-ASIP_)/V_P_; background polygenic heritability, h^2^_POLY_ = V_POLY_/V_P_; *STX17* mutation heritability, h^2^_STX17_ = V_STX17_/V_P_; and *ASIP* heritability, h^2^_ASIP_ = V_ASIP_/V_P_. For grey level, vitiligo grade and speckling grade, α_ASIP_ was not significant, so we assumed V_ASIP_ was zero. The analyses were performed for three scenarios: the first and third ignored *STX17* and *ASIP* effects, using the full data set and a data set containing *GG* animals only; the second applied the full model as described above for melanoma and excluded ASIP (because not significant) for the other traits.

Bivariate models were run for all pairs of traits applying the single trait model specifications from above. Genetic (*r*_POLY_) and environmental (*r*_e_) correlations between traits *x_1_* and *x_2_* were calculated as *r*_POLY_ = Cov_POLY__(*x_1_*, *x_2_*)/[(V_POLY__ *x_1_*)(V_POLY__ *x_2_*)]^0.5^ and *r*_e_ = Cov_e_(*x_1_*, *x_2_*)/[(V_e_ *x_1_*)(V_e_ *x_2_*)]*^0.5^*, while phenotypic correlations (*r*_P_) were calculated as r_P_ = r_POLY_(h^2^_x1_)^0.5^(h^2^_x2_)^0.5^+r_e_[(1−h^2^_x1_)]^0.5^[(1−h^2^_x2_)]^0.5^. Contour plots with 95% confidence intervals, which present breeding values standardized to a mean of zero and a standard deviation of one, were created for two traits obtained from bivariate animal models with respect to *STX17* genotype. These plots illustrate the decomposition of genetic correlations into additive polygenic background effects and effects of a single mutation. More detailed derivations of quantitative genetic parameters are provided in [39].
